# Crystal structure and Hirshfeld surface analysis of 6-imino-8-(4-methyl­phen­yl)-1,3,4,6-tetra­hydro-2*H*-pyrido[1,2-*a*]pyrimidine-7,9-dicarbo­nitrile

**DOI:** 10.1107/S2056989024002500

**Published:** 2024-03-21

**Authors:** Farid N. Naghiyev, Victor N. Khrustalev, Mehmet Akkurt, Ekaterina V. Dobrokhotova, Ajaya Bhattarai, Ali N. Khalilov, İbrahim G. Mamedov

**Affiliations:** a Department of Chemistry, Baku State University, Z. Khalilov str. 23, Az, 1148, Baku, Azerbaijan; b Peoples’ Friendship University of Russia (RUDN University), Miklukho-Maklay St. 6, Moscow 117198, Russian Federation; c N. D. Zelinsky Institute of Organic Chemistry RAS, Leninsky Prosp. 47, Moscow, 119991, Russian Federation; d Department of Physics, Faculty of Sciences, Erciyes University, 38039 Kayseri, Türkiye; e Department of Chemistry, M.M.A.M.C (Tribhuvan University) Biratnagar, Nepal; f "Composite Materials" Scientific Research Center, Azerbaijan State Economic University (UNEC), H. Aliyev str. 135, Az 1063, Baku, Azerbaijan; Institute of Chemistry, Chinese Academy of Sciences

**Keywords:** crystal structure, 1,2-di­hydro­pyridine ring, 1,3-diazinane ring, hydrogen bonds, C–H⋯π inter­actions, Hirshfeld surface analysis

## Abstract

In the ten-membered 1,3,4,6-tetra­hydro-2*H*-pyrido[1,2-*a*]pyrimidine ring system of the title compound, C_17_H_15_N_5_, the 1,2-di­hydro­pyridine ring is essentially planar (r.m.s. deviation = 0.001 Å), while the 1,3-diazinane ring has a distorted twist-boat conformation. In the crystal, mol­ecules are linked by N—H⋯N and C—H⋯N hydrogen bonds, forming a three-dimensional network. In addition, C—H⋯π inter­actions form layers parallel to the (100) plane. Thus, crystal-structure cohesion is ensured. According to a Hirshfeld surface study, H⋯H (40.4%), N⋯H/H⋯N (28.6%) and C⋯H/H⋯C (24.1%) inter­actions are the most important contributors to the crystal packing.

## Abstract 2

In the crystal, mol­ecules are linked by N—H⋯N and C–H⋯N hydrogen bonds, forming a three-dimensional network. In addition, C—H⋯π inter­actions form layers parallel to the (100) plane. Thus, crystal-structure cohesion is ensured.

## 1. Chemical context

Heterocyclic compounds are crucial systems, both in terms of frequency of occurrence and consequential importance in different fields (Khalilov *et al.*, 2022 ▸; Akkurt *et al.*, 2023 ▸). Heterocyclic systems comprise all nucleic acids, alkaloids, vitamins, sugars, hormones, anti­biotics, other drugs, dyes, pesticides, and herbicides. There have been major developments in organic chemistry in recent years with recently developed heterocyclic systems for various research and commercial aims, especially in the pharmaceutical and chemical industries (Maharramov *et al.*, 2022 ▸; Erenler *et al.*, 2022 ▸). These compounds have found widespread applications in multiple branches of science, such as coordination chemistry (Gurbanov *et al.*, 2021 ▸; Mahmoudi *et al.*, 2021 ▸), medicinal chemistry (Askerova, 2022 ▸) and materials chemistry (Velásquez *et al.*, 2019 ▸; Afkhami *et al.*, 2019 ▸). Pyrido[1,2-*a*]pyrimidines are simple bicyclic ring systems that contain a nitro­gen-bridgehead condensed pyrimidine motif. These derivatives are used for a large range of applications, as well as drugs, ligands, catalysts, materials, *etc* (Maharramov *et al.*, 2021 ▸, Sobhi & Faisal, 2023 ▸). Functionalized pyrido[1,2-*a*]pyrimidines exhibit various biological activities, such as anti­cancer, anti­oxidant, cytotoxic, anti-inflammatory, herbicidal, pesticidal, anti­bacterial (Atalay *et al.*, 2022 ▸; Donmez & Turkyılmaz, 2022 ▸). In medical practice, pyrido[1,2-*a*]pyrimidines are used as tranquilizers, anti-ulcerative agents, anti­allergics, anti-asthmatics, analgesics, anti­psychotics, protective gastrointestinal, neurotropic, stress-protecting compounds, and anti-HIV agents (Elattar *et al.*, 2017 ▸). As a result of the wide application of these systems, the efficient and regioselective development of pyrido[1,2-*a*]pyrimidines has attracted a lot of attention. Thus, in the framework of our studies in heterocyclic chemistry (Naghiyev *et al.*, 2020 ▸, 2021 ▸, 2022 ▸), herein we report the crystal structure and Hirshfeld surface analysis of the title compound, 6-imino-8-(4-methyl­phen­yl)-1,3,4,6-tetra­hydro-2*H*-pyrido[1,2-*a*]pyrimidine-7,9-dicarbo­nitrile. 
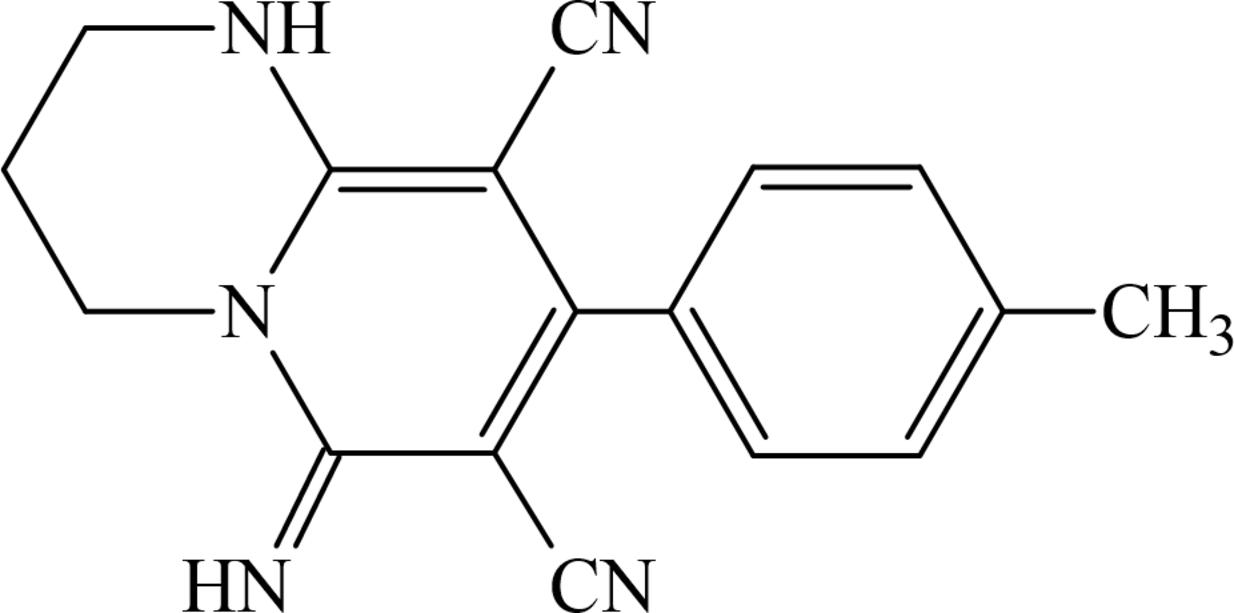


## 2. Structural commentary

**Figure 1:**
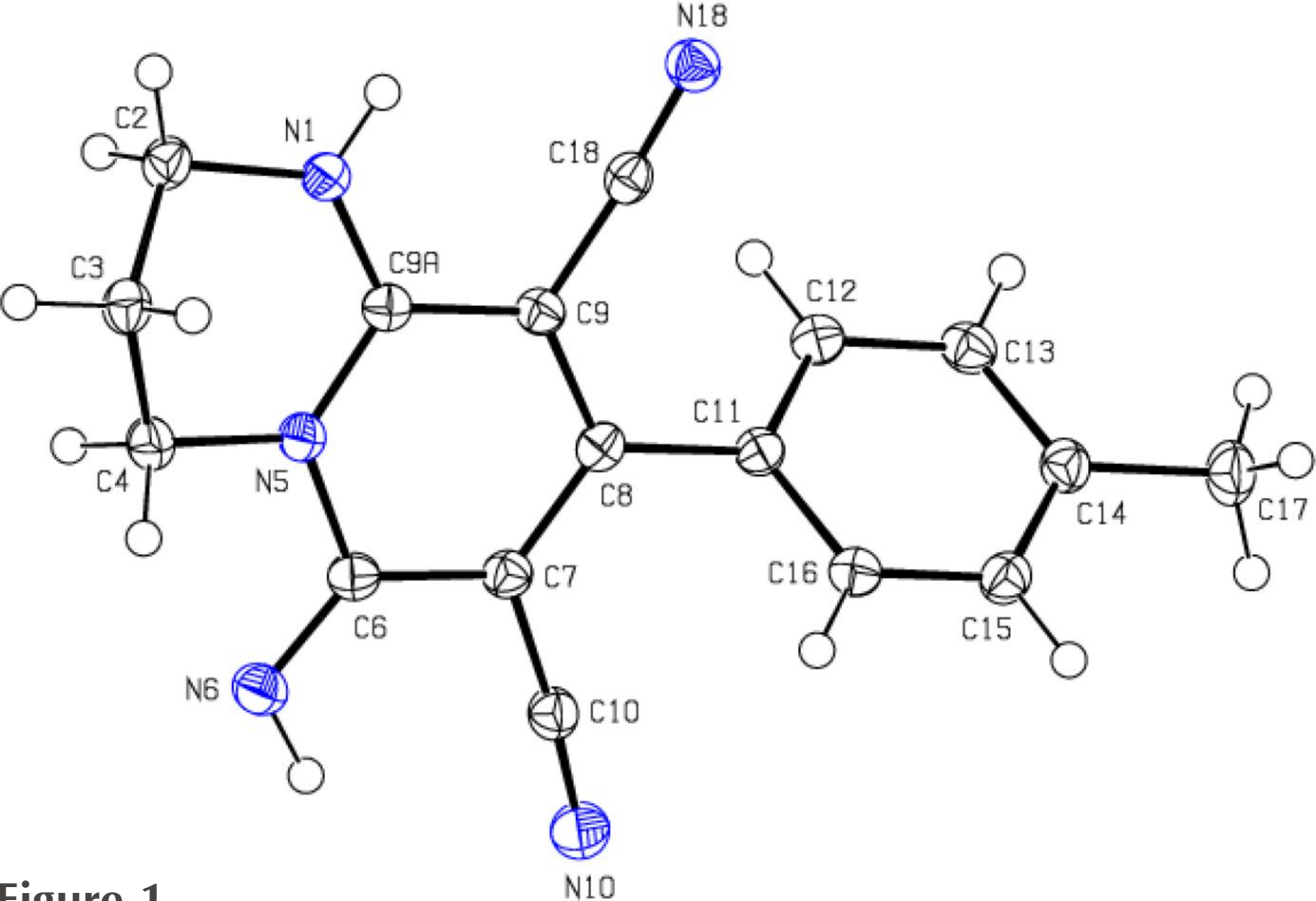
The mol­ecular structure of the title compound, showing the atom labelling and displacement ellipsoids drawn at the 50% probability level.

As seen in Fig. 1 ▸, in the ten-membered 1,3,4,6-tetra­hydro-2*H*-pyrido[1,2-*a*]pyrimidine ring system (N1/N5/C2–C9/C9*A*) of the title compound, the 1,2-di­hydro­pyridine ring (C11–C16) is essentially planar (r.m.s. deviation = 0.001 Å), while the 1,3-diazinane ring (N1/N5/C2–C4/C9*A*) has a distorted twist-boat conformation [puckering parameters (Cremer & Pople, 1975 ▸): *Q*_T_ = 0.5085 (14) Å, θ = 122.41 (15)° and φ = 281.45 (17)°]. The plane of the 1,2-di­hydro­pyridine ring makes dihedral angles of 11.49 (6) and 47.52 (6)°, respectively, with the mean plane of the 1,3-diazine and benzene rings. The angle between the mean plane of the 1,3-diazine and benzene rings is 41.40 (6)°. The torsion angles C11—C8—C7—C10, C11—C8—C9—C18 and C8—C7—C6—N6 are 4.73 (19), −4.83 (18) and −179.04 (13) °, respectively. The geometric parameters of the title compound are normal and comparable to those of the related compounds listed in the *Database survey* section.

## 3. Supra­molecular features and Hirshfeld surface analysis

**Figure 2:**
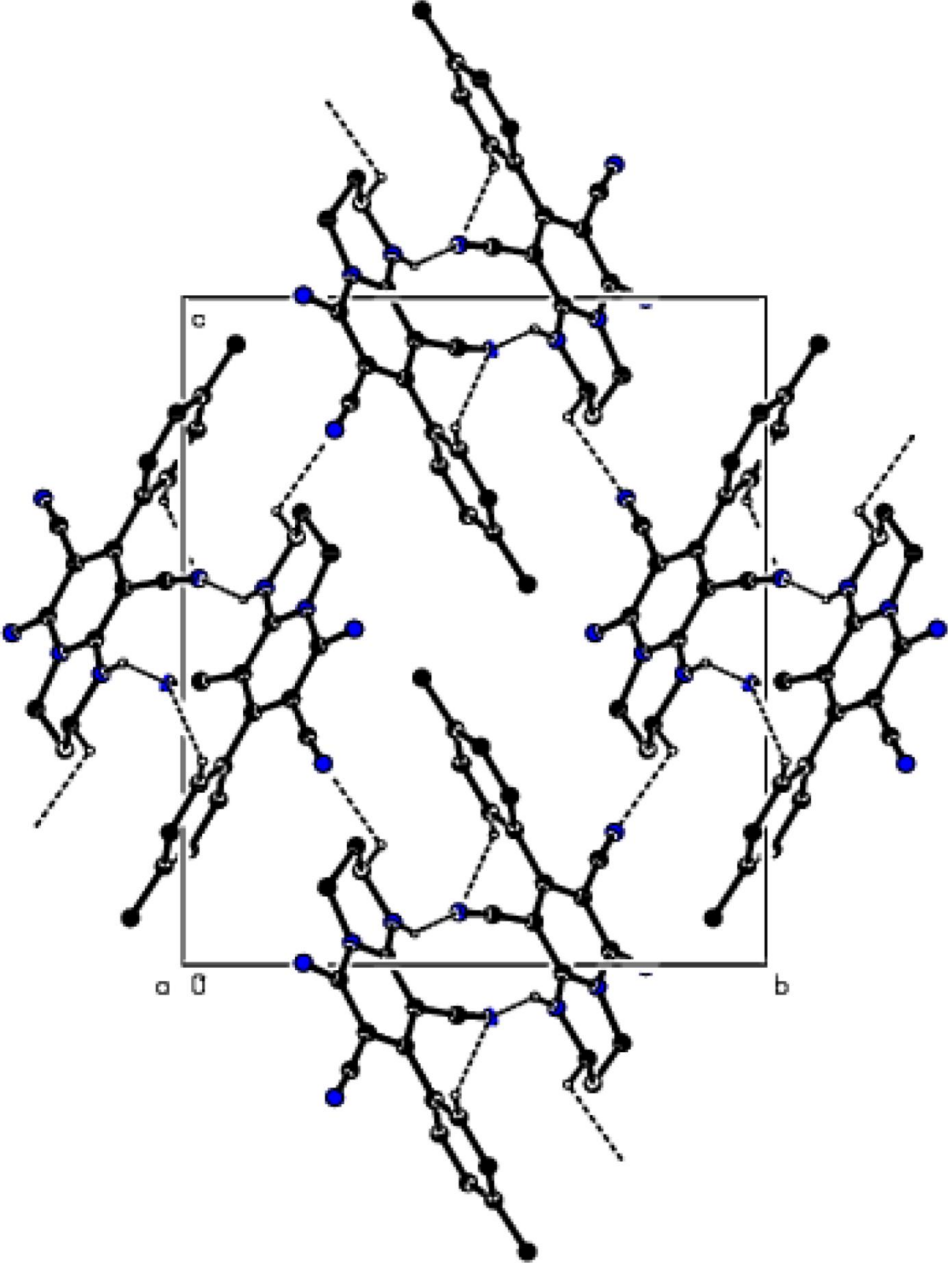
The packing viewed along the *a*-axis of the title compound with N—H⋯N and C—H⋯N hydrogen bonds shown as dashed lines.

**Figure 4:**
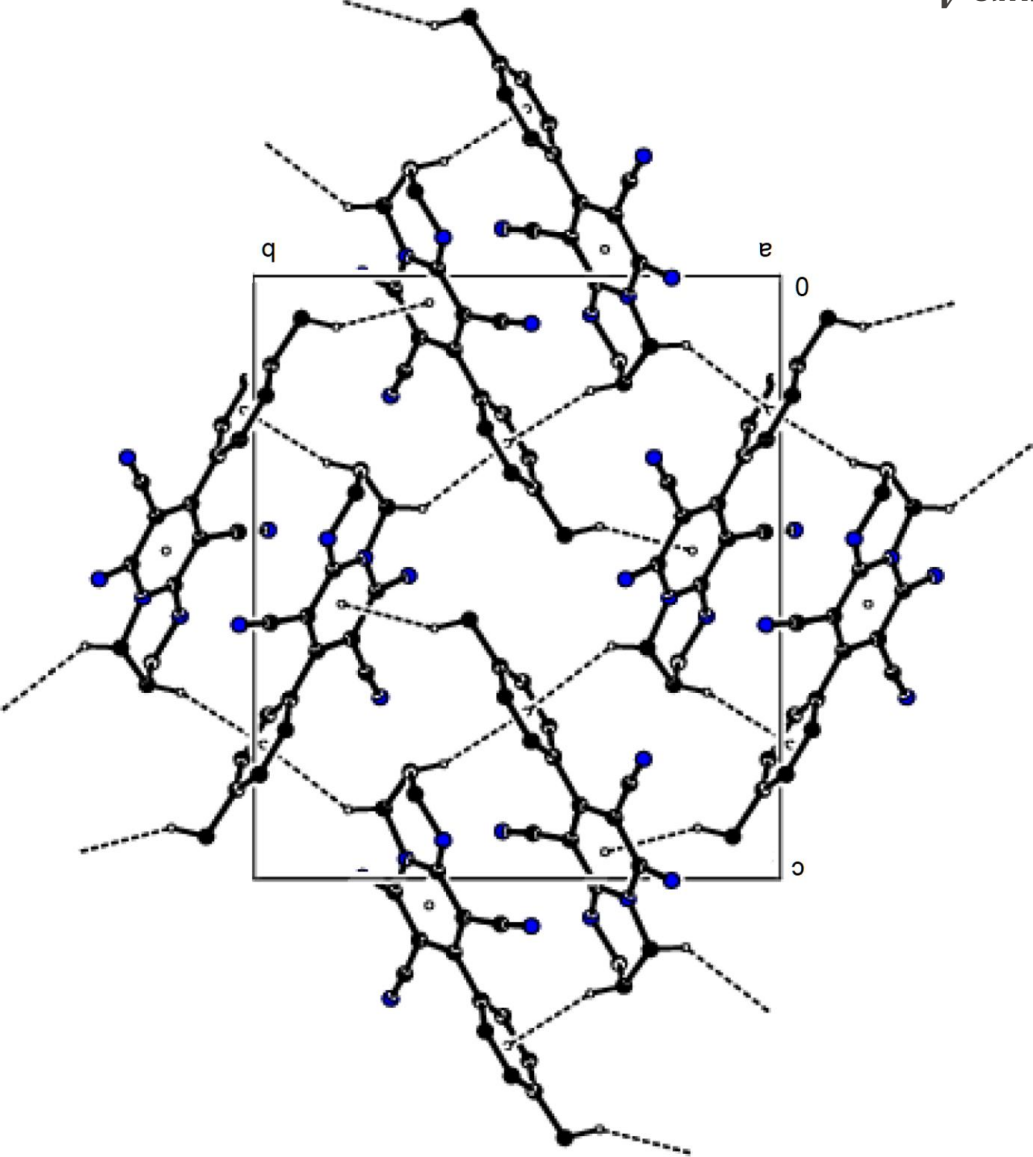
A view of the packing along the *a*-axis of the title compound with C—H⋯π inter­actions shown as dashed lines.

In the crystal, mol­ecules are linked by N—H⋯N and C–H⋯N hydrogen bonds, forming a three-dimensional network (Table 1 ▸; Figs. 2 ▸ and 3 ▸). In addition, C—H⋯π inter­actions form layers parallel to the (100) plane (Table 1 ▸; Figs. 4 ▸ and 5 ▸). Thus, crystal-structure cohesion is ensured.

**Figure 6:**
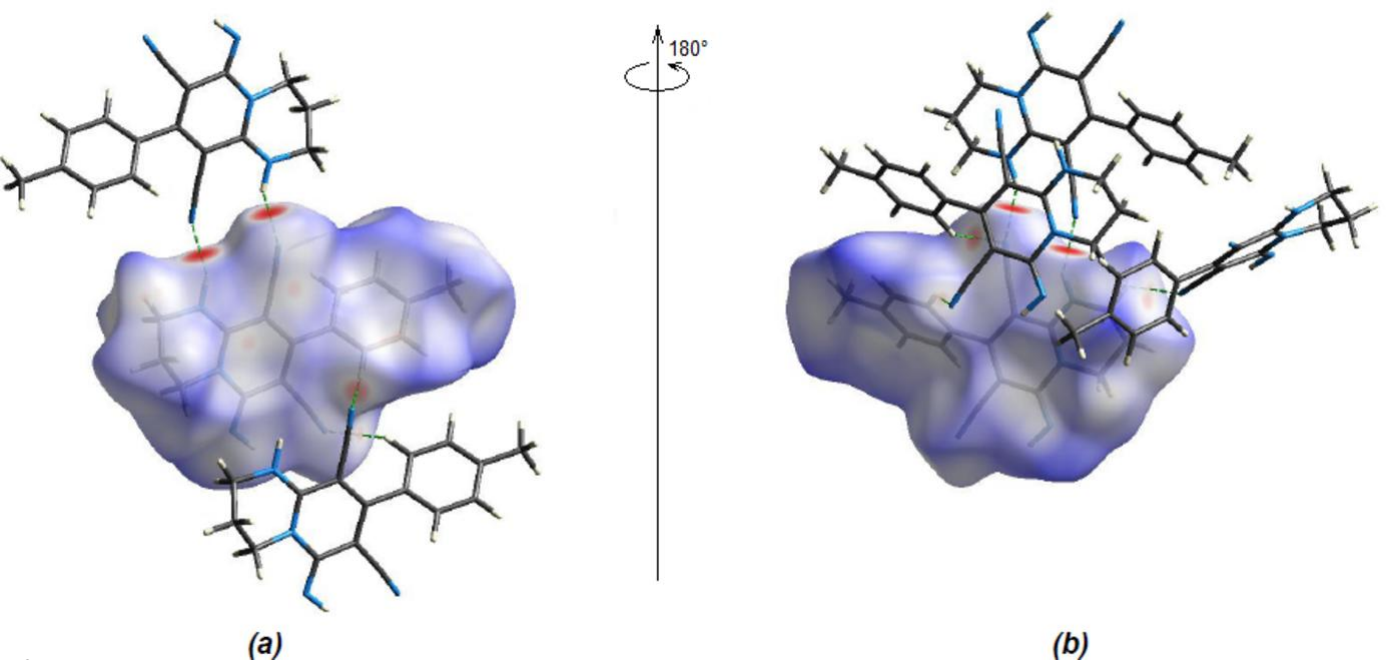
(*a*) Front and (*b*) back sides of the three-dimensional Hirshfeld surface of the title compound mapped over *d_norm_*.

**Figure 7:**
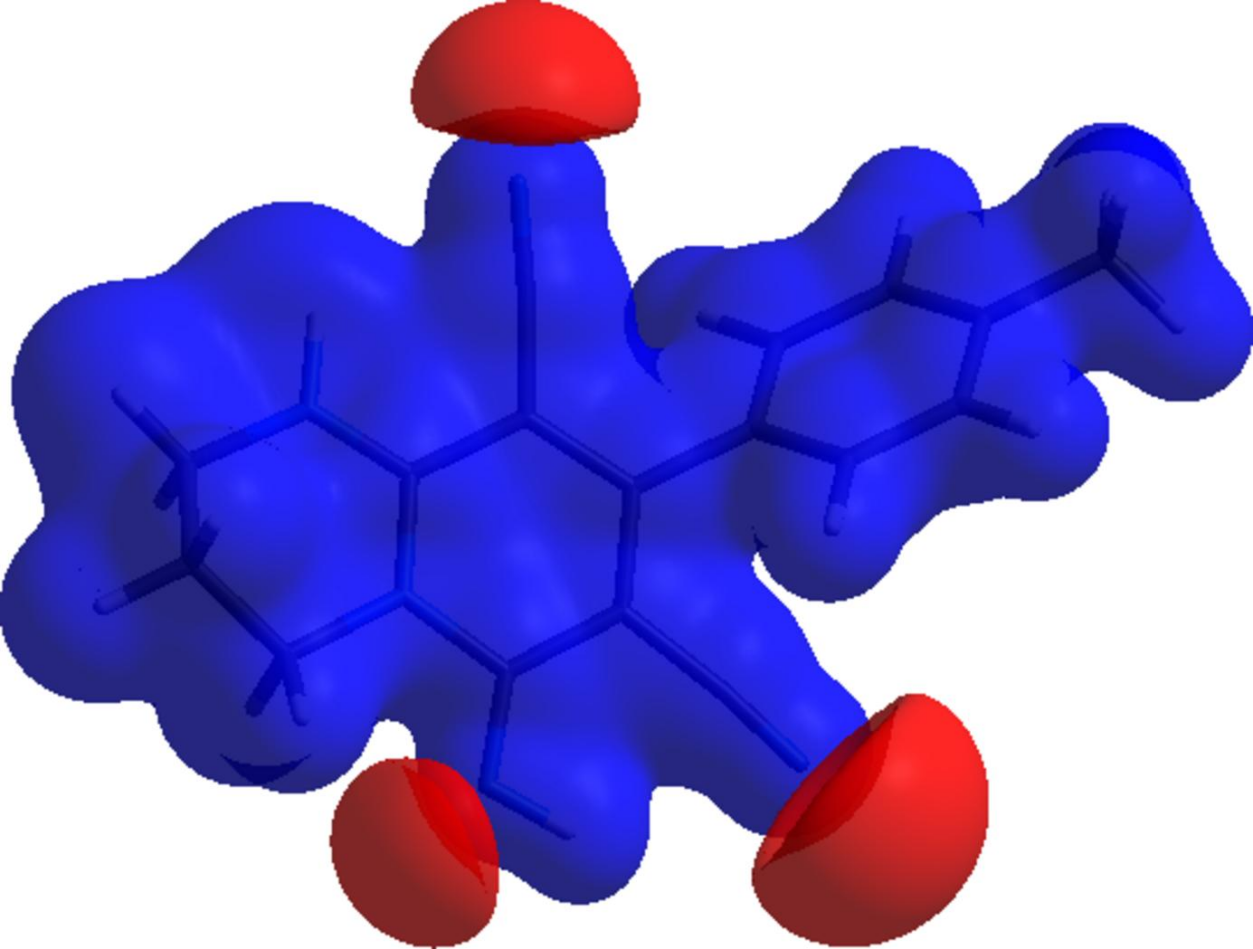
View of the electrostatic potential energy surface of the title compound calculated using the STO-3G basis set at the Hartree–Fock level of theory.

In order to qu­antify the inter­molecular inter­actions in the crystal, *Crystal Explorer 17.5* (Spackman *et al.*, 2021 ▸) was used to generate Hirshfeld surfaces and two-dimensional fingerprint plots. The Hirshfeld surfaces mapped over *d*_norm_ are shown in Fig. 6 ▸. The bright-red spots indicate their roles as respective donors and/or acceptors; they also appear as blue and red regions corresponding to positive and negative potentials on the electrostatic potential energy surface (Fig. 7 ▸).

**Figure 8:**
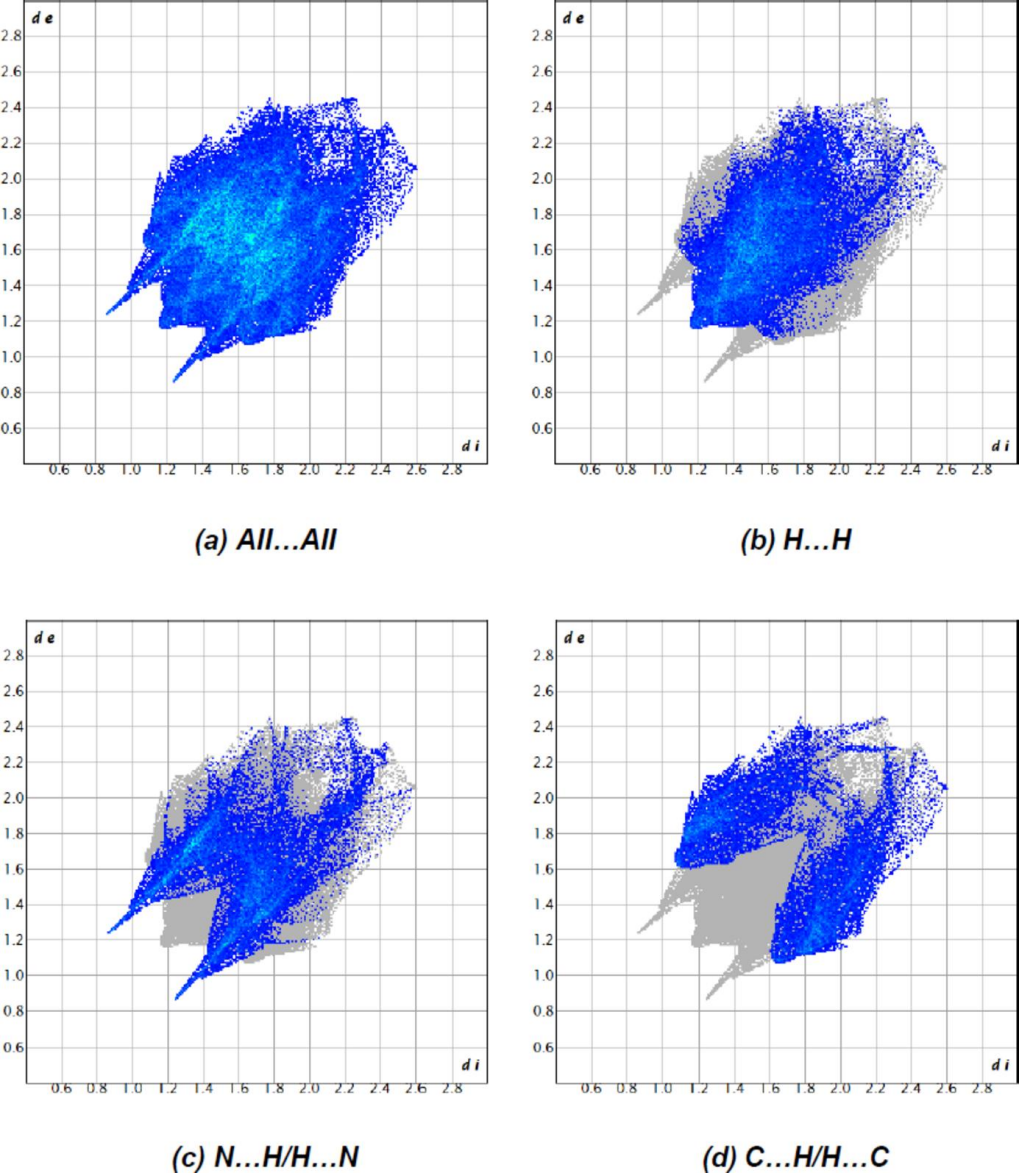
The two-dimensional fingerprint plots, showing (*a*) all inter­actions, and delineated into (*b*) H⋯H, (*c*) N⋯H/H⋯N and (*d*) C⋯H/H⋯C inter­actions. [*d*_e_ and *d*_i_ represent the distances from a point on the Hirshfeld surface to the nearest atoms outside (external) and inside (inter­nal) the surface, respectively].

The most important inter­atomic contact is H⋯H as it makes the highest contribution to the crystal packing (40.4%, Fig. 8 ▸ *b*). The other major contributors are the N⋯H/H⋯N (28.6%, Fig. 8 ▸ *c*) and C⋯H/H⋯C (24.1%, Fig. 8 ▸ *d*) inter­actions. Other, smaller contributions are made by N⋯H/H⋯N (2.8%), C⋯C (2.7%) and N⋯N (1.4%) inter­actions.

## 4. Database survey

Five related compounds, which also have the 1,3,4,6-tetra­hydro- 2*H*-pyrido[1,2-*a*]pyrimidine ring system seen in the title compound, were found in a search of the Cambridge Structural Database (CSD version 5.42, update of November 2020; Groom *et al.*, 2016 ▸): CSD refcode IQEFOC (Naghiyev *et al.*, 2021 ▸), VAMBET (Khodjaniyazov & Ashurov, 2016 ▸), HECLUZ (Khodjaniyazov *et al.*, 2017 ▸), LEGLIU (Chen *et al.*, 2012 ▸) and KUTPEV (Samarov *et al.*, 2010 ▸).

In IQEFOC, inter­molecular N—H⋯N and C—H⋯N hydrogen bonds form mol­ecular sheets parallel to the (110) and ( 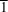 10) planes, crossing each other. Adjacent mol­ecules are further linked by C—H⋯π inter­actions, which form zigzag chains propagating parallel to [100]. In the crystal of VAMBET, mol­ecules are linked *via* C— H⋯O and C—H⋯N hydrogen bonds, forming layers parallel to (101). In the crystal of HECLUZ, hydrogen bonds with 16-membered ring and three chain motifs are generated by N—H⋯N and N—H⋯O contacts. The amino group is located close to the nitro­gen atoms, forming hydrogen bonds with 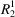 (4) and 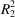 (12) graph-set motifs. This amino group also forms a hydrogen bond with the C=O oxygen atom of a mol­ecule translated parallel to [100], which links the mol­ecules into 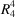 (16) rings. Hydrogen-bonded chains are formed along [100] by alternating 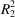 (12) and 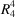 (16) rings. These chains are stabilized by inter­molecular π–π stacking inter­actions observed between the pyridine and pyrimidine rings. In LEGLIU, the mol­ecular structure is built up from two fused six-membered rings and one seven-membered ring linked through a spiro C atom. The crystal packing is stabilized by inter­molecular N—H⋯O hydrogen bonds between the two N—H groups and the ketone O atoms of the neighbouring mol­ecules. In KUTPEV, water mol­ecules are mutually O—H⋯O hydrogen bonded and form infinite chains propagating parallel to [010]. Neighbouring chains are linked by the quinazoline mol­ecules by means of O—H⋯O=C hydrogen bonds, forming a diperiodic network.

## 5. Synthesis and crystallization

A solution of 2-(4-methyl­benzyl­idene)malono­nitrile (6 mmol) and malono­nitrile (6.1 mmol) in methanol (35 mL) was stirred for 10 min. Then 1,3-di­amino­propane (5.3 mol) was added to the reaction mixture and stirred for 72 h. Then 25 mL of methanol were removed from the reaction mixture, which was left overnight. The precipitated crystals were separated by filtration and recrystallized from an ethanol/water (1:1) solution (m.p. 501–502 K, yield 36%).

^1^H NMR (300 MHz, DMSO-*d*_6_, ppm.): 1.98 (*m*, 2H, CH_2_); 2.39 (*s*, 3H, CH3-Ar); 3.42 (*t*, 2H, CH_2_, ^3^ *J*_H–H_ = 6.9); 3.93 (*t*, 2H, CH_2_, ^3^ *J*_H–H_ = 6.9); 4.14 (*s*, 1H, CH-Ar); 6.41 (*s*, 2H, NH_2_); 7.30–7.42 (*m*, 4H, 4Ar-H); 7.70 (*s*, 1H, NH). ^13^C NMR (75 MHz, DMSO-*d*_6_, ppm.): 19.46 (CH_2_), 21.01 (Ar-CH_3_), 38.36 (Ar-CH), 39.64 (NCH2), 41.59 (NCH2), 51.64 (=C_quat._), 57.25 (=C_quat._), 120.70 (CN), 121.12 (CN), 128.14 (2CH_arom._), 128.98 (2CH_arom._), 134.92 (C_arom._), 140.77 (C_arom._), 151.15 (=C_quat._), 152.36 (=C_quat._).

## 6. Refinement

Crystal data, data collection and structure refinement details are summarized in Table 2 ▸. All C-bound H atoms were placed at calculated positions and refined using a riding model, with C—H = 0.95–0.99 Å, and with *U*_iso_(H) = 1.2 or 1.5*U*_eq_(C). The N-bound H atoms were located in difference-Fourier maps [N1—H1 = 0.894 (17) Å, N6—H6 = 0.944 (18) Å] and refined with *U*_iso_(H) = 1.2*U*_eq_(N).

## Supplementary Material

Crystal structure: contains datablock(s) I. DOI: 10.1107/S2056989024002500/nx2006sup1.cif

Structure factors: contains datablock(s) I. DOI: 10.1107/S2056989024002500/nx2006Isup2.hkl

Supporting information file. DOI: 10.1107/S2056989024002500/nx2006Isup3.cml

CCDC reference: 2340712

Additional supporting information:  crystallographic information; 3D view; checkCIF report

## Figures and Tables

**Figure 3:**
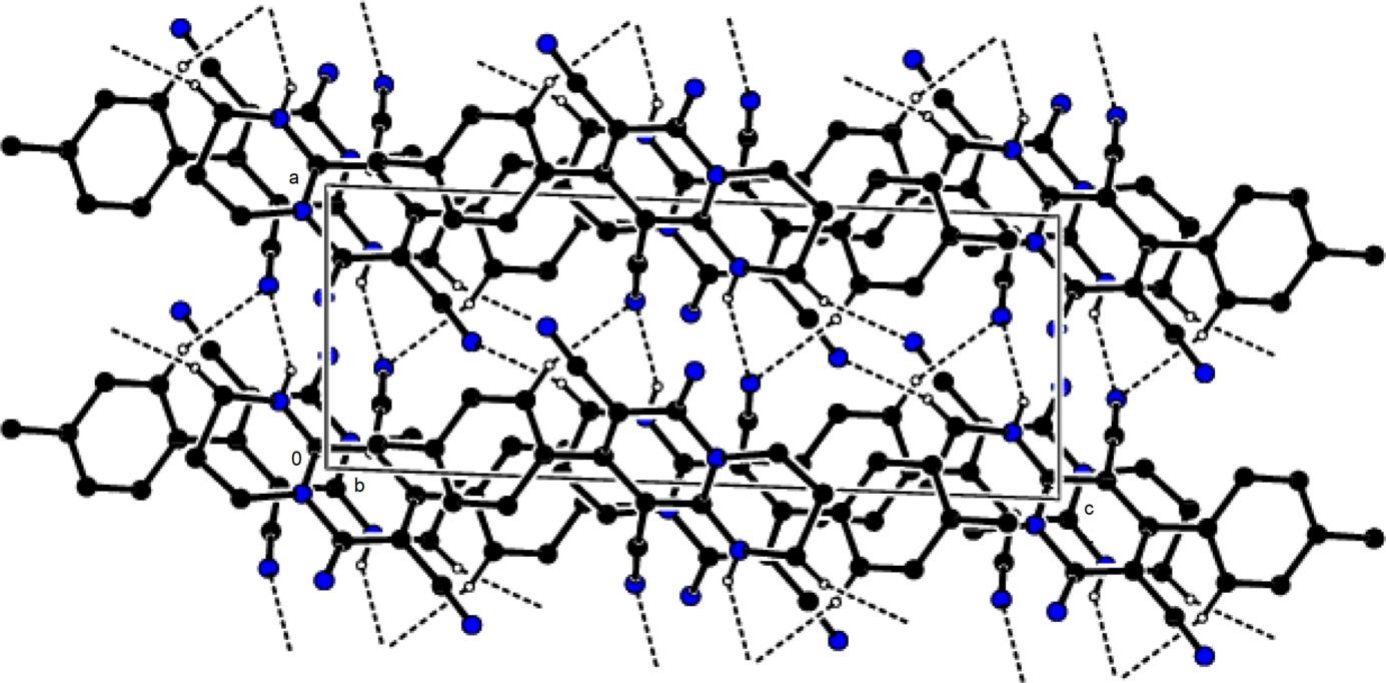
The packing viewed along the *b*-axis of the title compound with N—H⋯N and C—H⋯N hydrogen bonds shown as dashed lines.

**Figure 5:**
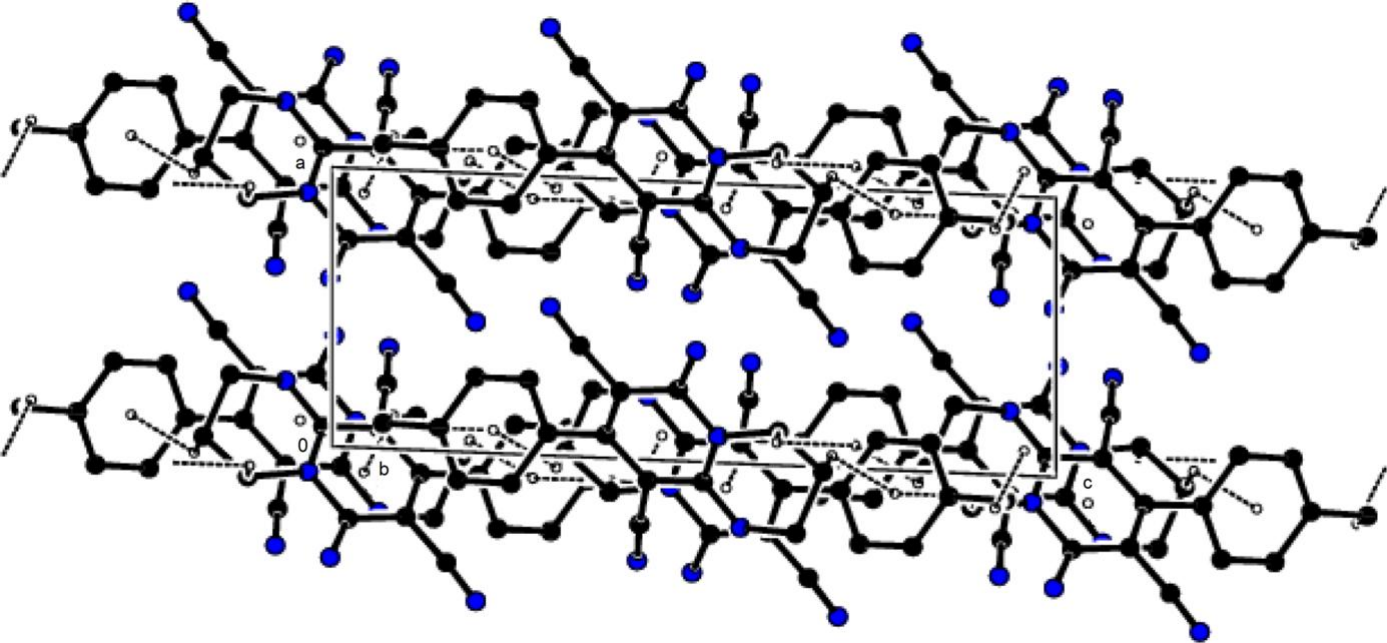
A view of the packing along the *b*-axis of the title compound with C—H⋯π inter­actions shown as dashed lines.

**Table 1:** Hydrogen-bond geometry (Å, °) *Cg*2 and *Cg*3 are the centroids of the N5/C6–C9/C9*A* and C11–C16 rings, respectively.

*D*—H⋯*A*	*D*—H	H⋯*A*	*D*⋯*A*	*D*—H⋯*A*
N1—H1⋯N18^i^	0.892 (18)	2.229 (18)	3.0308 (16)	149.3 (14)
C2—H2*B*⋯N10^ii^	0.99	2.60	3.1574 (18)	116
C16—H16⋯N18^iii^	0.95	2.51	3.3592 (17)	149
C3—H3*B*⋯*Cg*3^iv^	0.99	2.77	3.5553 (15)	136
C4—H4*B*⋯*Cg*3^v^	0.99	2.88	3.6750 (14)	138
C17—H17*C*⋯*Cg*2^vi^	0.98	2.88	3.6306 (16)	134

Symmetry codes: (i) 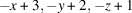 ; (ii) 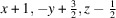 ; (iii) 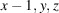 ; (iv) 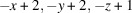 ; (v) 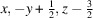 ; (vi) 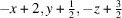 .

**Table 2:** Experimental details

Crystal data	
Chemical formula	C_17_H_15_N_5_
*M*_r_	289.34
Crystal system, space group	Monoclinic, *P*2_1_/*c*
Temperature (K)	100
*a*, *b*, *c* (Å)	6.2459 (4), 14.1480 (9), 16.2111 (11)
β (°)	92.435 (7)
*V* (Å^3^)	1431.23 (16)
*Z*	4
Radiation type	Synchrotron, λ = 0.74500 Å
μ (mm^−1^)	0.09
Crystal size (mm)	0.13 × 0.03 × 0.01

Data collection	
Diffractometer	Rayonix SX165 CCD
Absorption correction	Multi-scan (*SCALA*; Evans, 2006 ▸)
*T*_min_, *T*_max_	0.981, 0.989
No. of measured, independent and observed [*I* > 2σ(*I*)] reflections	16784, 3956, 3119
*R*_int_	0.049
(sin θ/λ)_max_ (Å^−1^)	0.692

Refinement	
*R*[*F* ^2^ > 2σ(*F* ^2^)], *wR*(*F* ^2^), *S*	0.045, 0.113, 1.04
No. of reflections	3956
No. of parameters	206
H-atom treatment	H atoms treated by a mixture of independent and constrained refinement
Δρ_max_, Δρ_min_ (e Å^−3^)	0.30, −0.24

Computer programs: *Marccd* (Doyle, 2011 ▸), *iMosflm* (Battye *et al.*, 2011 ▸), *SHELXT2014/5* (Sheldrick, 2015*a* ▸), *SHELXL2019/3* (Sheldrick, 2015*b* ▸), *ORTEP-3 for Windows* (Farrugia, 2012 ▸) and *PLATON* (Spek, 2020 ▸).

## supplementary crystallographic information

### Crystal data

**Table:**

C_17_H_15_N_5_	*F*(000) = 608
*M**_r_* = 289.34	*D*_x_ = 1.343 Mg m^−^^3^
Monoclinic, *P*2_1_/*c*	Synchrotron radiation, λ = 0.74500 Å
*a* = 6.2459 (4) Å	Cell parameters from 600 reflections
*b* = 14.1480 (9) Å	θ = 2.6–26.0°
*c* = 16.2111 (11) Å	µ = 0.09 mm^−^^1^
β = 92.435 (7)°	*T* = 100 K
*V* = 1431.23 (16) Å^3^	Needle, yellow
*Z* = 4	0.13 × 0.03 × 0.01 mm

### Data collection

**Table:**

Rayonix SX165 CCD diffractometer	3119 reflections with *I* > 2σ(*I*)
/f scan	*R*_int_ = 0.049
Absorption correction: multi-scan (Scala; Evans, 2006)	θ_max_ = 31.0°, θ_min_ = 2.6°
*T*_min_ = 0.981, *T*_max_ = 0.989	*h* = −8→8
16784 measured reflections	*k* = −19→19
3956 independent reflections	*l* = −22→22

### Refinement

**Table:**

Refinement on *F*^2^	0 restraints
Least-squares matrix: full	Hydrogen site location: mixed
*R*[*F*^2^ > 2σ(*F*^2^)] = 0.045	H atoms treated by a mixture of independent and constrained refinement
*wR*(*F*^2^) = 0.113	*w* = 1/[σ^2^(*F*_o_^2^) + (0.0455*P*)^2^ + 0.6432*P*] where *P* = (*F*_o_^2^ + 2*F*_c_^2^)/3
*S* = 1.04	(Δ/σ)_max_ < 0.001
3956 reflections	Δρ_max_ = 0.30 e Å^−^^3^
206 parameters	Δρ_min_ = −0.24 e Å^−^^3^

### Special details

**Table:**

Geometry. All esds (except the esd in the dihedral angle between two l.s. planes) are estimated using the full covariance matrix. The cell esds are taken into account individually in the estimation of esds in distances, angles and torsion angles; correlations between esds in cell parameters are only used when they are defined by crystal symmetry. An approximate (isotropic) treatment of cell esds is used for estimating esds involving l.s. planes.

### Fractional atomic coordinates and isotropic or equivalent isotropic displacement parameters (Å^2^)

**Table:**

	*x*	*y*	*z*	*U*_iso_*/*U*_eq_	
N1	1.22846 (18)	0.85962 (8)	0.43598 (6)	0.0203 (2)	
H1	1.338 (3)	0.8968 (12)	0.4519 (10)	0.024*	
C2	1.2417 (2)	0.80620 (10)	0.35940 (7)	0.0218 (2)	
H2A	1.303447	0.742845	0.370787	0.026*	
H2B	1.334227	0.839650	0.320792	0.026*	
C3	1.0165 (2)	0.79718 (10)	0.32220 (7)	0.0217 (2)	
H3A	1.018198	0.760667	0.270134	0.026*	
H3B	0.957031	0.860657	0.309545	0.026*	
C4	0.8787 (2)	0.74716 (9)	0.38329 (8)	0.0220 (3)	
H4A	0.726345	0.751480	0.364155	0.026*	
H4B	0.918397	0.679453	0.385898	0.026*	
N5	0.90562 (17)	0.78939 (8)	0.46706 (6)	0.0188 (2)	
C6	0.7437 (2)	0.76717 (9)	0.52209 (7)	0.0196 (2)	
N6	0.60252 (19)	0.70637 (8)	0.49720 (7)	0.0246 (2)	
H6	0.504 (3)	0.6963 (13)	0.5390 (11)	0.030*	
C7	0.7591 (2)	0.81478 (9)	0.60171 (7)	0.0191 (2)	
C8	0.91651 (19)	0.88052 (9)	0.62207 (7)	0.0181 (2)	
C9	1.07340 (19)	0.89846 (9)	0.56422 (7)	0.0184 (2)	
C9A	1.07054 (19)	0.84860 (9)	0.48773 (7)	0.0177 (2)	
C10	0.6013 (2)	0.78674 (9)	0.65782 (8)	0.0218 (3)	
N10	0.4674 (2)	0.75999 (9)	0.69863 (8)	0.0291 (3)	
C11	0.91976 (19)	0.93141 (9)	0.70229 (7)	0.0185 (2)	
C12	1.1058 (2)	0.93959 (9)	0.75279 (7)	0.0206 (2)	
H12	1.235238	0.911116	0.736637	0.025*	
C13	1.1016 (2)	0.98949 (10)	0.82682 (8)	0.0224 (3)	
H13	1.228854	0.994184	0.860790	0.027*	
C14	0.9150 (2)	1.03264 (9)	0.85215 (8)	0.0219 (3)	
C15	0.7295 (2)	1.02285 (9)	0.80184 (8)	0.0220 (3)	
H15	0.599979	1.050941	0.818303	0.026*	
C16	0.7307 (2)	0.97293 (9)	0.72833 (7)	0.0204 (2)	
H16	0.602065	0.966821	0.695323	0.025*	
C17	0.9088 (2)	1.09051 (11)	0.92991 (8)	0.0285 (3)	
H17A	1.047423	1.086173	0.960301	0.043*	
H17B	0.795831	1.066382	0.964407	0.043*	
H17C	0.878970	1.156666	0.915642	0.043*	
C18	1.2316 (2)	0.96948 (9)	0.57532 (7)	0.0190 (2)	
N18	1.36293 (18)	1.02717 (8)	0.57859 (7)	0.0228 (2)	

### Atomic displacement parameters (Å^2^)

**Table:**

	*U*^11^	*U*^22^	*U*^33^	*U*^12^	*U*^13^	*U*^23^
N1	0.0197 (5)	0.0226 (5)	0.0188 (5)	−0.0025 (4)	0.0026 (4)	−0.0023 (4)
C2	0.0240 (6)	0.0227 (6)	0.0189 (5)	−0.0004 (5)	0.0036 (4)	−0.0017 (5)
C3	0.0249 (6)	0.0227 (6)	0.0174 (5)	0.0001 (5)	0.0010 (4)	−0.0015 (4)
C4	0.0236 (6)	0.0229 (6)	0.0195 (5)	−0.0021 (5)	0.0007 (4)	−0.0035 (4)
N5	0.0193 (5)	0.0199 (5)	0.0173 (4)	−0.0008 (4)	0.0011 (4)	−0.0010 (4)
C6	0.0186 (5)	0.0207 (6)	0.0195 (5)	−0.0004 (5)	0.0011 (4)	0.0016 (4)
N6	0.0240 (5)	0.0264 (6)	0.0236 (5)	−0.0055 (5)	0.0023 (4)	−0.0015 (4)
C7	0.0195 (5)	0.0194 (6)	0.0186 (5)	−0.0006 (5)	0.0021 (4)	0.0010 (4)
C8	0.0182 (5)	0.0188 (6)	0.0173 (5)	0.0027 (5)	0.0004 (4)	0.0013 (4)
C9	0.0179 (5)	0.0191 (6)	0.0181 (5)	−0.0012 (5)	0.0000 (4)	0.0003 (4)
C9A	0.0170 (5)	0.0175 (5)	0.0184 (5)	0.0014 (4)	−0.0004 (4)	0.0019 (4)
C10	0.0235 (6)	0.0216 (6)	0.0203 (5)	−0.0012 (5)	0.0015 (5)	−0.0013 (4)
N10	0.0315 (6)	0.0284 (6)	0.0278 (6)	−0.0052 (5)	0.0082 (5)	−0.0006 (5)
C11	0.0195 (6)	0.0188 (5)	0.0173 (5)	−0.0009 (5)	0.0016 (4)	0.0008 (4)
C12	0.0204 (6)	0.0214 (6)	0.0200 (5)	0.0002 (5)	0.0013 (4)	0.0020 (4)
C13	0.0216 (6)	0.0246 (6)	0.0208 (6)	−0.0029 (5)	−0.0011 (4)	0.0009 (5)
C14	0.0256 (6)	0.0210 (6)	0.0191 (5)	−0.0024 (5)	0.0023 (5)	−0.0009 (4)
C15	0.0218 (6)	0.0211 (6)	0.0233 (6)	0.0018 (5)	0.0029 (4)	−0.0003 (5)
C16	0.0190 (6)	0.0215 (6)	0.0207 (5)	−0.0008 (5)	0.0003 (4)	0.0009 (4)
C17	0.0328 (7)	0.0293 (7)	0.0232 (6)	0.0009 (6)	−0.0002 (5)	−0.0077 (5)
C18	0.0204 (5)	0.0213 (6)	0.0154 (5)	0.0022 (5)	0.0004 (4)	−0.0001 (4)
N18	0.0223 (5)	0.0250 (5)	0.0211 (5)	−0.0020 (5)	−0.0003 (4)	−0.0004 (4)

### Geometric parameters (Å, º)

**Table:**

N1—C9A	1.3310 (16)	C8—C11	1.4859 (16)
N1—C2	1.4587 (16)	C9—C18	1.4155 (17)
N1—H1	0.894 (17)	C9—C9A	1.4259 (16)
C2—C3	1.5123 (18)	C10—N10	1.1524 (18)
C2—H2A	0.9900	C11—C12	1.3974 (17)
C2—H2B	0.9900	C11—C16	1.4002 (17)
C3—C4	1.5150 (18)	C12—C13	1.3936 (17)
C3—H3A	0.9900	C12—H12	0.9500
C3—H3B	0.9900	C13—C14	1.3929 (19)
C4—N5	1.4865 (15)	C13—H13	0.9500
C4—H4A	0.9900	C14—C15	1.3947 (18)
C4—H4B	0.9900	C14—C17	1.5050 (18)
N5—C9A	1.3586 (16)	C15—C16	1.3855 (17)
N5—C6	1.4120 (16)	C15—H15	0.9500
C6—N6	1.2847 (17)	C16—H16	0.9500
C6—C7	1.4555 (17)	C17—H17A	0.9800
N6—H6	0.944 (18)	C17—H17B	0.9800
C7—C8	1.3833 (17)	C17—H17C	0.9800
C7—C10	1.4252 (17)	C18—N18	1.1567 (17)
C8—C9	1.4080 (17)		
			
C9A—N1—C2	123.11 (11)	C9—C8—C11	120.77 (11)
C9A—N1—H1	117.8 (10)	C8—C9—C18	123.04 (11)
C2—N1—H1	118.7 (11)	C8—C9—C9A	120.44 (11)
N1—C2—C3	107.31 (10)	C18—C9—C9A	116.40 (11)
N1—C2—H2A	110.3	N1—C9A—N5	119.41 (11)
C3—C2—H2A	110.3	N1—C9A—C9	120.53 (11)
N1—C2—H2B	110.3	N5—C9A—C9	120.06 (11)
C3—C2—H2B	110.3	N10—C10—C7	174.99 (14)
H2A—C2—H2B	108.5	C12—C11—C16	118.66 (11)
C2—C3—C4	108.88 (10)	C12—C11—C8	122.20 (11)
C2—C3—H3A	109.9	C16—C11—C8	119.13 (11)
C4—C3—H3A	109.9	C13—C12—C11	120.05 (12)
C2—C3—H3B	109.9	C13—C12—H12	120.0
C4—C3—H3B	109.9	C11—C12—H12	120.0
H3A—C3—H3B	108.3	C14—C13—C12	121.50 (12)
N5—C4—C3	111.35 (10)	C14—C13—H13	119.2
N5—C4—H4A	109.4	C12—C13—H13	119.2
C3—C4—H4A	109.4	C13—C14—C15	117.94 (11)
N5—C4—H4B	109.4	C13—C14—C17	122.48 (12)
C3—C4—H4B	109.4	C15—C14—C17	119.56 (12)
H4A—C4—H4B	108.0	C16—C15—C14	121.27 (12)
C9A—N5—C6	122.49 (10)	C16—C15—H15	119.4
C9A—N5—C4	121.90 (10)	C14—C15—H15	119.4
C6—N5—C4	115.55 (10)	C15—C16—C11	120.55 (12)
N6—C6—N5	116.82 (11)	C15—C16—H16	119.7
N6—C6—C7	127.35 (12)	C11—C16—H16	119.7
N5—C6—C7	115.81 (11)	C14—C17—H17A	109.5
C6—N6—H6	109.5 (11)	C14—C17—H17B	109.5
C8—C7—C10	122.59 (11)	H17A—C17—H17B	109.5
C8—C7—C6	122.82 (11)	C14—C17—H17C	109.5
C10—C7—C6	114.58 (11)	H17A—C17—H17C	109.5
C7—C8—C9	118.05 (11)	H17B—C17—H17C	109.5
C7—C8—C11	121.18 (11)	N18—C18—C9	175.26 (13)
			
C9A—N1—C2—C3	38.85 (16)	C6—N5—C9A—N1	173.71 (11)
N1—C2—C3—C4	−59.02 (13)	C4—N5—C9A—N1	−9.17 (18)
C2—C3—C4—N5	48.56 (14)	C6—N5—C9A—C9	−6.41 (18)
C3—C4—N5—C9A	−14.66 (16)	C4—N5—C9A—C9	170.71 (11)
C3—C4—N5—C6	162.65 (11)	C8—C9—C9A—N1	−174.37 (11)
C9A—N5—C6—N6	−176.32 (12)	C18—C9—C9A—N1	9.52 (17)
C4—N5—C6—N6	6.39 (17)	C8—C9—C9A—N5	5.75 (18)
C9A—N5—C6—C7	2.43 (17)	C18—C9—C9A—N5	−170.36 (11)
C4—N5—C6—C7	−174.86 (11)	C7—C8—C11—C12	132.01 (13)
N6—C6—C7—C8	−179.04 (13)	C9—C8—C11—C12	−48.44 (17)
N5—C6—C7—C8	2.36 (18)	C7—C8—C11—C16	−48.17 (17)
N6—C6—C7—C10	2.2 (2)	C9—C8—C11—C16	131.37 (13)
N5—C6—C7—C10	−176.36 (11)	C16—C11—C12—C13	−0.95 (18)
C10—C7—C8—C9	175.72 (12)	C8—C11—C12—C13	178.86 (12)
C6—C7—C8—C9	−2.91 (18)	C11—C12—C13—C14	−0.42 (19)
C10—C7—C8—C11	−4.73 (19)	C12—C13—C14—C15	1.33 (19)
C6—C7—C8—C11	176.65 (11)	C12—C13—C14—C17	−177.21 (13)
C7—C8—C9—C18	174.72 (11)	C13—C14—C15—C16	−0.88 (19)
C11—C8—C9—C18	−4.83 (18)	C17—C14—C15—C16	177.71 (12)
C7—C8—C9—C9A	−1.12 (18)	C14—C15—C16—C11	−0.48 (19)
C11—C8—C9—C9A	179.33 (11)	C12—C11—C16—C15	1.40 (18)
C2—N1—C9A—N5	−4.15 (18)	C8—C11—C16—C15	−178.42 (12)
C2—N1—C9A—C9	175.97 (11)		

### Hydrogen-bond geometry (Å, º)

*Cg*2 and *Cg*3 are the centroids of the N5/C6–C9/C9*A* and C11–C16 rings, respectively.

**Table:**

*D*—H···*A*	*D*—H	H···*A*	*D*···*A*	*D*—H···*A*
N1—H1···N18^i^	0.892 (18)	2.229 (18)	3.0308 (16)	149.3 (14)
C2—H2*B*···N10^ii^	0.99	2.60	3.1574 (18)	116
C16—H16···N18^iii^	0.95	2.51	3.3592 (17)	149
C3—H3*B*···*Cg*3^iv^	0.99	2.77	3.5553 (15)	136
C4—H4*B*···*Cg*3^v^	0.99	2.88	3.6750 (14)	138
C17—H17*C*···*Cg*2^vi^	0.98	2.88	3.6306 (16)	134

Symmetry codes: (i) −*x*+3, −*y*+2, −*z*+1; (ii) *x*+1, −*y*+3/2, *z*−1/2; (iii) *x*−1, *y*, *z*; (iv) −*x*+2, −*y*+2, −*z*+1; (v) *x*, −*y*+1/2, *z*−3/2; (vi) −*x*+2, *y*+1/2, −*z*+3/2.
